# RNA interference as a gene silencing therapy for mutant *MYOC *protein in primary open angle glaucoma

**DOI:** 10.1186/1746-1596-4-46

**Published:** 2009-12-16

**Authors:** Mao Li, Jianjiang Xu, Xueli Chen, Xinghuai Sun

**Affiliations:** 1 Department of Ophthalmology and Vision Science, Eye and Ear Nose Throat Hospital, Shanghai Medical School, Fudan University, No. 83, Fenyang Road, Shanghai, PR China

## Abstract

**Background:**

Primary open-angle glaucoma (POAG) is the most common form of glaucoma which is an irreversible blind leading disease and lacks effective remedies. In recent years, POAG has been linked to the gene *MYOC *encoding myocilin that has been identified to harbor causal mutations. A variety of studies show that the mutant myocilin acts by gain of function. The mutant *MYOC *protein induces endoplasmic reticulum (ER) stress and the resultant unfolded protein response (UPR) induces apoptosis in the trabecular meshwork cells, which then leads to an increase in resistance to aqueous humor outflow, elevated intraocular pressure (IOP), and, ultimately, glaucoma. Culturing human trabecular meshwork (HTM) cells at a condition facilitating protein folding promotes secretion of mutant myocilin, normalizes cell morphology and reverses cell lethality.

**Presentation of the Hypothesis:**

We speculate that a complete elimination of mutant myocilin expression in trabecular meshwork cells is safe and that gives the possibility of avoiding the POAG phenotype.

**Testing the Hypothesis:**

We propose RNA interference (RNAi) as a gene silencing therapy to eliminate the mutant myocilin proteins in the trabecular meshwork cells, either in a mutation-dependent or mutation-independent way due to the different engineering of the small interfering (si) RNA.

**Implications of the Hypothesis:**

The RNAi strategy can reverse the pathological process of trabecular meshwork cells and thus treat the POAG caused by myocilin gene mutation. This strategy can also be applicable to many protein-misfolding diseases caused by gain-of-function mutant proteins.

## Background

Glaucoma is a group of progressive optic neuropathies that have in common a slow progressive degeneration of retinal ganglion cells and their axons, resulting in a distinct appearance of the optic disc and a concomitant pattern of visual loss[1]. Without adequate treatment, glaucoma can progress to irreversible visual disability and eventual blindness [1]. Of the many types of glaucoma, primary open angle glaucoma (POAG) is perhaps the most common, particularly in populations of African ancestry and European [2-4]. In most cases of POAG, increased resistance to the outflow of aqueous humor results in a rise in intraocular pressure (IOP), which eventually leads to loss of retinal ganglion cells[5]. Though Increased IOP has been proven to be the only treatable risk factor for glaucoma, the biological basis of the disease is not yet fully understood[1]. This may underlies that the present treatment of POAG directed at lowering IOP[6] does not seem to halt all cases of progression [7-10]. To date, six loci (GLC1A-E) have been linked to POAG alone. Among them, the gene *MYOC *encoding myocilin has been identified as harboring causal mutations, which are responsible for 3-4% adult-onset POAG cases [11]. This warrants the gene therapy which holds the promise for curing the disease. Current investigations of gene therapy mainly focus on the transfer and expression of genes encoding IOP-lowering, neuroprotective gene products, and/or, wound healing inhibitors[12], but do not really emphasize a specific elimination of the causal mutant proteins. Here we propose RNA interference (RNAi) as gene silencing therapy for complete elimination of mutant myocilin from human trabecular meshwork (HTM) cells.

## Presentation of Hypothesis

The human myocilin gene encodes a 504 amino acid glycoprotein expressed extra- or intracellularly in almost every ocular tissue[13,14] but predominantly in the trabecular meshwork [15]which is a tissue provides major resistance to the aqueous humor outflow pathway, and the tissue involved in elevated IOP associated with glaucoma. However, *MYOC*-null individuals (no matter mice [16] or human beings [17,18] have normal eyes and intraocular pressure. These individuals are both viable and fertile as well [16-18]. Overexpression of wild type (WT) *MYOC *in transgenic mice does not lead to any glaucomatous phenotype[19,20]. All these facts suggest that haploinsufficiency of *MYOC *is not a critical mechanism in the pathogenesis of POAG [16-20].

A variety of recent studies show that the mutant myocilin acts in the HTM cells by gain of function[16,21-27]. It was observed that, in culture media, very little to no mutant myocilin associated with the development of glaucoma was secreted out of the HTM cells, while WT and polymorphism variant myocilin were secreted normally[26]. Further investigations found that the disease-causing myocilin mutants were actually misfolded, were highly aggregation-prone and accumulated in large aggregates or formed heteromeric complexes with WT myocilin in the endoplasmic reticulum (ER)[5,22,24,28]. The ER is responsible for the synthesis, modification and delivery of correctly folded proteins to their proper target sites. Increased expression of mutant, folding-incompetent proteins causes ER stress and an ER stress response, called the unfolded protein response(UPR). The UPR is an adaptive mechanism to return the ER to its normal physiological state by upregulating the ER folding capacity and down-regulating the biosynthetic load of the ER. When the UPR does not remedy the stress situation, apoptosis is initiated in higher eukaryotic organisms, presumably to eliminate unhealthy cells [29]. In the case of *MYOC*-associated POAG, the aggregates induced the unfolded protein response proteins BiP and phosphorylated endoplasmic reticulum-localized eukaryotic initiation factor-2_ kinase (PERK) with the subsequent activation of caspases 12 and 3 and expression of C/EBP homologous protein (CHOP)/GADD153, leading to abnormal HTM cell morphology and cell apoptosis[24,28]. The progressive loss of HTM cells due to apoptosis should accordingly diminish the phagocytotic capacity of the remaining TM cell population to clean the outflow drainage and eventually results in humor outflow resistance and elevated IOP[24]. This is consist with the electron microscopic findings from biopsies of POAG patients showing a reduction of TM cells as well as thickened trabeculae and accumulation of sheath-derived plaques [30-33].

Since the mutant myocilin induces ER stress and eventually leads to the apoptosis of HTM cells and hence the pathogenesis of *Myoc- *associated glaucoma, a complete elimination of mutant myocilin in HTM cells may disrupt or even partly reverse the disease. In support of this proposal are two major previous investigation findings: (1) Culturing HTM cells at a condition facilitating protein folding promotes secretion of mutant myocilin, normalizes cell morphology and reverses cell lethality[5], and (2) *MYOC*-null individuals have no glaucomatous phenotype and are both viable and fertile as well [16-18].

## Testing the Hypothesis

To eliminate the mutant myocilin in HTM cells, RNAi can be used as a powerful gene silencing method [34-37], either in a mutation-dependent [38] or a mutation-independent way [39]. In mutation dependent RNAi, a customized double-stranded small interfering (si) RNA targeting a mutant sequence is synthesized and conducted by a plasmid to the HTM cell expressing the very mutant myocilin. The siRNA can be engineered specifically to suppress the mutant alleles differing from WT alleles as little as only a single nucleotide[38], so that the WT myocilin can express normally in the heterozygotes without being insulted. However, myocilin-associated POAG is intragenic heterogeneous [40] and customized siRNA inhibition targeting large numbers of individual mutations would be costly. A more economical approach of mutation-independent silencing will avoid this drawback. In this approach, siRNA complementary to the untranslated regions (UTRs) of the target mRNA is conducted to the HTM cell and hence both the WT and mutant alleles would be suppressed. Moreover, a replacement WT myocilin gene with modified UTRs that is sparing from the suppression can be generated and conducted into the cell, allowing the expression of WT alleles while suppressing that of the mutant ones simultaneously[39]. In the case of *MYOC-*associated POAG, the insertion of the replacement WT myocilin gene may be unnecessary for suppression of myocilin gene totally limited to HTM cells might not lead to pathological phenotypes. If such a suppression without inserting the replacement WT gene is safe, the mutation-independent strategy would probably be the best choice among the above proposals.

## Implications of the Hypothesis

In this article, we propose RNAi as a gene silencing therapy for the *MYOC*-associated POAG. It has been demonstrated by abundant researches [5,22,24,28] that mutant myocilin proteins are insufficiently folded and accumulate in the ER, inducing UPR with subsequently activated cell apoptosis. We hypothesize that if these mutant proteins are suppressed by siRNA mediated RNAi, the cell lesion would be reversed. To the best of our knowledge, our idea of treating POAG by inhibiting the disease causing gene has not been stated before. Those investigated strategies of gene therapy for glaucoma do not aim at the disease causing mutants, but focus on inducing genes compensating for the defects [12], which are actually the modifications of contemporary treatments at the gene level. Compared with this, the RNAi strategy we propose here is more specific to the etiological factor and would likely to be more effective. Shinohara et al [41]proposed a hypothesis of treating the retinitis pigmentosa (RP) by silencing the mutant rhodopsin in a allele-specific way. However, since MYOC-null individuals have been reported to be healthy, we suspect that totally suppressing the myocilin without inserting a replacement WT myocilin to HTM cells would be safe and economically feasible.

ER retention of mutant proteins has been implicated in the pathogenesis of many ER storage diseases other than POAG, including some inherited neurodegenerative disorders like Alzheimer's disease [29]. The RNAi strategy aiming at eliminating the misfolded mutant proteins can also be applied to these disorders. Problems to be solved are the safety and efficiency of RNAi. A highly efficient siRNA should be chosen from a variety of candidates and the side effects should be observed with caution by a series of investigations before it can be carried out clinically.

## Competing interests

The authors declare that they have no competing interests.

## Authors' contributions

ML conceived the hypothesis and drafted the manuscript; JX conceived the hypothesis and gave modifications of the manuscript; XC involved in drafting the manuscript; XS revised the manuscript critically and gave final approval of the version to be published
